# Breast lipofilling as a treatment for breast hypoplasia in Becker naevus syndrome: a case report

**DOI:** 10.1093/jscr/rjaa583

**Published:** 2021-01-29

**Authors:** Natasha Christodoulides, Gerard A Kelly, Sean T O’Sullivan

**Affiliations:** Department of Plastic and Reconstructive Surgery, Cork University Hospital, Wilton, Cork, Ireland; Department of Plastic and Reconstructive Surgery, Cork University Hospital, Wilton, Cork, Ireland; Department of Plastic and Reconstructive Surgery, Cork University Hospital, Wilton, Cork, Ireland

## Abstract

A Becker’s naevus is a rare, pigmented, cutaneous hamartoma, which when associated with other cutaneous or musculoskeletal anomalies is termed Becker naevus syndrome. Female patients commonly seek medical attention for breast hypoplasia. Here, we describe our experience in the surgical management of unilateral breast hypoplasia in a patient with Becker naevus syndrome, using high-volume autologous fat grafting. This is, to our knowledge, the second report in the literature describing the aforementioned management technique in this patient cohort.

## INTRODUCTION

A Becker’s naevus a rare, pigmented, cutaneous hamartoma, with irregular boarders, located predominantly on the anterior trunk or scapular region, first described by Becker in 1949 [1]. Although reports in the literature described the association of a Becker’s naevus with unilateral breast hypoplasia, hypertrichosis or musculoskeletal anomalies, it is not until 1997 that Happle and Koopman proposed the term `Becker naevus syndrome’ [2, 3]. Female patients usually present seeking medical treatment for breast hypoplasia and/or the thoracic naevus [4]. Breast hypoplasia is classically treated with silicone implants [4]. As these patients often present at a young age, treatment with silicone implants could result in significant complications in the long-term including rupture, capsular contracture and breast implant associated anaplastic large cell lymphoma [5]. The patient may therefore be subjected to further procedures later in life for implant exchange or removal [6]. Here, we describe our own experience in the management of a patient with Becker naevus syndrome presenting with unilateral breast hypoplasia treated with breast lipofilling. This is an alternative approach to management with only one published case series in the literature [4].

## CASE PRESENTATION

A 25-year-old woman with no significant co-morbidities presented to our outpatients clinic with right breast hypoplasia. Overlying the hypoplastic breast on the right thoraco-mammary region, an irregular, hyperpigmented macular lesion was noted. The underlying pectoralis major muscle, latissimus dorsi, nipple and areola were normal. According to the patient, the hypermelanosis had been present since puberty with no significant change in the size or degree of pigmentation. No other anomaly was detected and the patient had no family history of note. A clinical diagnosis of Becker naevus syndrome was made.

Following discussion surrounding the possible treatment options, the decision was made to manage the breast hypoplasia with liposuction and autologous fat grafting. The procedure was carried out in two sessions, 17 months apart.

The donor site was infused with 500 ml tumescent solution of Hartmann’s, 1.25 mg/ml chirocaine, 1 ml of 1:1000 adrenaline and 1500 IU hyaluronidase. Fat was harvested from the flanks and lower abdomen using power-assisted liposuction.

The harvested fat was processed to eliminate tumescent fluid, blood, cell fragments and oil. Lipoaspirate was processed using the Revolve system (LifeCell, Bridgewater, NJ). Fat was infiltrated into the hypoplastic breast using a blunt Coleman infiltration cannula, from deep to superficial while retracting the cannula. A 300 ml concentrate was infiltrated in the first procedure, whereas a 150 ml concentrate was infiltrated in the second procedure.

**Figure 1:**
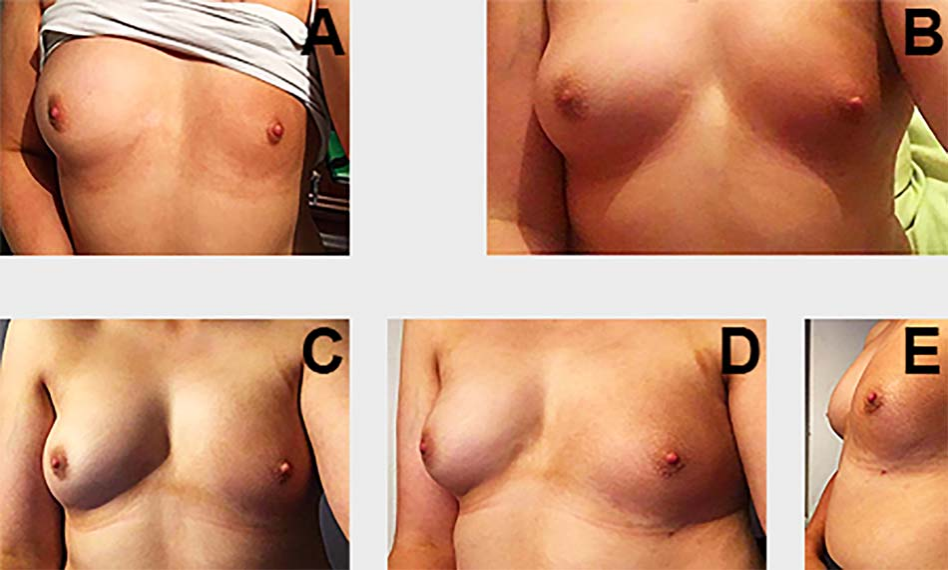
(**A**) Before the first procedure; (B) Result after the one session of lipofilling; (**C–E**) Result following two sessions of lipofilling with notable improvement in breast symmetry and pigmentation of the nevus (pictures taken in a mirror).

The injection points and liposuction areas were closed using a 4-0 absorbable suture. The areas were dressed with omnistrips and mepore. A supportive bra was applied. No post-operative complications were noted. Considerable improvement in breast symmetry and degree of pigmentation was evident on follow-up with a high degree of patient satisfaction (Fig. 1).

## DISCUSSION

Becker naevus syndrome is equally prevalent between males and females, most often presenting at puberty, with breast hypoplasia being the most frequent reason for patient concern [7].

Whilst Becker’s naevus in association with breast hypoplasia is well documented, there is only one study in the literature describing the role of autologous fat grafting in its management [4]. Although implant reconstruction may initially provide favourable cosmetic effects, emerging data regarding long-term complications may result in patients seeking alternative methods of management [5, 6].

Since its introduction, lipofilling has become increasingly popular in breast augmentation and asymmetry [8]. Advantages include easy availability of donor tissue, absence of a scar and short recovery time [8]. Although historically thought to yield unpredictable results, technique evolution has led to increased use of autologous fat grafting by plastic surgeons worldwide with consistently excellent results [9, 10]. Although Becker naevus syndrome is rare, it is important to consult patients on all available options for breast augmentation, including fat grafting as a safe treatment option with minimal side effects [9].

## CONCLUSIONS

Treatment for breast hypoplasia associated with a Becker’s naevus is complex, with no recommendations or management guidelines from international societies of plastic and reconstructive surgery. Here, we present autologous fat grafting as a safe, effective option for management, with excellent cosmetic results.

## CONFLICT OF INTEREST STATEMENT

None declared.

## FUNDING

None.
